# Forging Online Community Among People in Recovery From Substance Use: Natural Language Processing and Deep-Learning Analysis of The Phoenix App User-Generated Data

**DOI:** 10.2196/68438

**Published:** 2025-12-19

**Authors:** Danny Valdez, Katie M Heinrich, Beth Collinson, Aspen Streetman, Zach Sloan

**Affiliations:** 1 Department of Applied Health Science, Indiana University, Bloomington, IN, United States; 2 Department of Research and Evaluation, The Phoenix, 54 Newmarket Sq, Boston, MA, 02118, United States, 1 8084579525; 3 Department of Pediatrics, Vanderbilt University Medical Center, Nashville, TN, United States; 4 The Phoenix, Boston, MA, United States

**Keywords:** community, mental health, mHealth, mobile health, social support, substance use

## Abstract

**Background:**

Mobile apps are powerful tools for promoting and sustaining healthy behaviors, including supporting diverse recovery pathways from substance use, including alcohol use disorder. Indeed, prior research strongly supports the notion that social connection through mobile platforms, supplemented by an in-person interaction, is vital in helping individuals strengthen their recovery and improve overall well-being and mental health. However, research into the digital footprints of mobile app users, as a strategy to assess app usage experiences in a recovery context, is lacking.

**Objective:**

This study utilizes a dataset from The Phoenix app, a social media platform specifically designed for individuals impacted by substance use, including those in or seeking recovery, to identify core uses of the app, including how it is leveraged by members from a thematic and emotional valence context.

**Methods:**

We applied natural language processing and deep learning methods to analyze a random sample of 19,685 posts. Analyses included the Bidirectional Encoder Representation from Transformers topic modeling tool to generate themes and a Valence Aware Dictionary and Sentiment Reasoner sentiment analysis to approximate emotional tone and mood from posts ranging from highly negative (−0.99) to highly positive (0.99).

**Results:**

After removing duplicate and nonsensical posts, we retained a final sample size of 17,617 posts. Bidirectional Encoder Representation from Transformers topic modeling tool identified 10 topics (coherence score=0.48) within 2 overarching themes: (1) those related to engaging app members through in-person and online interactions (7 topics) and (2) as a forum to discuss more serious topics pertaining to substance use and mental health recovery (3 topics). Overall, the topics revealed a distinct and recurring theme of community support. Valence Aware Dictionary and Sentiment Reasoner sentiment analysis was 0.44 (SD 0.42), indicating highly positive posts, with only 429 (2.4%) being highly negative.

**Conclusions:**

The study findings broadly show positive uses of The Phoenix app as a tool for social connections and community among people in recovery from substance use. With the high positive sentiment of posts, the app was distinct from other social media platforms (eg, X, Reddit, Facebook), which often feature a mix of highly positive and highly negative posts. Additional research is needed to confirm these results using a larger dataset and with comparative analysis of other recovery forums to contribute to the understanding of social media’s role and function in changing health-related behaviors.

## Introduction

### Background

Recovery is a behavioral health process among individuals affected by substance use and substance use disorders (SUDs) [1]. In 2012, the Substance Abuse and Mental Health Services Administration developed a new working definition of recovery, defining the process as “a…change through which individuals improve their health and wellness, live self-directed lives, and strive to reach their full potential” [2]. This definition shifts away from the traditional emphasis on abstinence along with symptom mitigation and reframes recovery as a holistic, multidimensional, and humanistic process [3]. By emphasizing overall well-being, the Substance Abuse and Mental Health Services Administration broadens the focus to include improvement in multiple life domains for individuals affected by substance use or other similar challenges. This shift underscores the importance of individualized and comprehensive recovery pathways that are accessible and not time-bound and foster supportive environments to promote recovery outcomes [4,5].

Emphasizing recovery is critical given the evolving US substance use crisis, which affects individuals across age groups and demographics [6]. As many as one-third of US adults reported an SUD in the past year, and the number of people facing substance-related challenges is growing substantially [7,8]. Yet, in the United States, most adults with SUD do not receive treatment [6] due to well-documented barriers to care that include cost, geographic inaccessibility, perceived stigma, and the availability of co-occurring disorder treatment [9-11]. Novel strategies that address this crisis with approaches that promote engagement in recovery by meeting people where they are in ways that are both accessible and intuitive are needed.

### Mobile Apps as Accessible Recovery-Promoting Tools

Growing evidence suggests that social media forums and smartphone apps may offer practical and cost-effective strategies for behavior change, which can significantly decrease substance use [12-14]. Some of the most frequently deployed behavior change strategies used on these forums and apps include self-monitoring, personalization, reminders [14], and online peer coaching [15]. These app features enable users to tailor the app’s functionality to meet their individual needs, resulting in personalized recovery plans, increased app use, prolonged engagement in recovery, and reductions in substance use [13,14,16,17].

Social media platforms, and their respective smartphone apps, also help facilitate social support for substance use and mental health recovery [18-21]. Previous research shows, for example, that online recovery forums on Reddit can provide a sense of belonging and provide practical advice for individuals with SUD through interactions with others who share lived experiences and peer coaches trained to discuss substance use virtually [19,22]. Additionally, hashtags and content related to recovery on platforms such as X and TikTok can raise awareness; reduce stigma; and connect individuals with resources, professional counseling, and other support networks [18,21]. Indeed, these virtual spaces allow people to share their experiences and seek guidance from others who have faced similar challenges, which can potentially support the engagement and connection necessary for promoting behavior change [23].

Other digital smartphone apps, which include closed social media–like platforms designed specifically for people in recovery, provide accessible, highly specific, and flexible opportunities for individuals to engage with recovery networks, often bypassing barriers known to impede care such as stigma [24]. Research likewise suggests that individuals are open to the idea of using social media or similar apps for recovery support, sometimes citing convenience and semianonymity in these spaces [25,26].

Recovery-specific digital platforms or apps—that is, InTheRooms and The Phoenix—have emerged as online resources beyond conventional social media designed to foster a community among people with lived experiences of substance use [23,25,27,28]. Although more research is needed on the long-term impacts of these apps, early indications suggest that they offer recovery-specific tools that provide emotional and practical support, paralleling the benefits of in-person interactions [29].

### Purpose

Individuals wanting to change behaviors may seek online and offline resources to initiate or sustain recovery from substance use and related challenges [30-32]. This study examines The Phoenix: A Sober Community, a nonprofit organization with a nationwide reach that developed a free mobile app (hereafter referred to as The Phoenix app, now known as NewForm). The app was designed to facilitate access to social support across geographical and temporal boundaries. It has since evolved into a robust platform that allows members and volunteers to connect based on shared interests or locations (eg, sober parents and guardians, veterans, arts, and crafts). It includes features such as live-streamed and in-person events, recovery journey tracking, volunteer coordination tools, and peer-to-peer communication.

As a social media app with a closed environment (ie, tailored for a specific purpose), understanding the app’s thematic and emotional digital ecosystem may offer additional insights into the role of digitally delivered peer- and volunteer-mediated interactions in promoting a safe and inclusive recovery environment. This study aims to identify the thematic and emotional climate of The Phoenix app using the in-the-moment interactions from anonymized app member data. Our study is guided by 3 research questions (RQs):

1. Using natural language processing and deep learning, what themes emerge from a corpus of posts originating from The Phoenix app?
2. What do compound sentiment analysis scores implicate regarding the personal and collective experiences of The Phoenix app members?
3. How can data mining strategies inform the future adaptation of The Phoenix app and member experiences?

The findings from this study will contribute to the growing body of evidence calling for directed and curated app experiences to promote behavior change in the context of substance use. In particular, the methods, or pipeline, undertaken in this study will also illustrate their effectiveness in highlighting app functionality along several critical domains, including language (ie, words and phrases used on mobile apps), emotions (ie, valence patterns used in mobile apps), and behaviors (ie, identifying observations in which recovery outcomes are salient). Collectively, this pipeline—which uses user-generated content provided by The Phoenix—will further document successful strategies necessary to sustain healthy digital environments for substance use recovery.

## Methods

### Data and Sample Allocation

#### Data

Data for this study were provided to the research team directly by The Phoenix and originated from The Phoenix app. The aim of our study was to test an unsupervised natural language processing (NLP) pipeline as part of a larger research project aimed at improving the functionality and usability of the app. These analyses are also intended to create a scaffold for building more refined machine learning and artificial intelligence algorithms trained to understand recovery-related language and needs.

By downloading and using the app, members agree to The Phoenix privacy policy, including consent to their anonymized data from the app for research purposes at The Phoenix. To uphold anonymity, posts derived from The Phoenix app are first deidentified by The Phoenix. During the anonymization phase, all personally identifiable information is removed, including a member’s name, location, and other personally identifiable information. Once data have been anonymized and deidentified, they are assigned a “Text ID” to facilitate easier comparisons and faster identification by the core data analysis and research teams. Once data have been completely deidentified, they are stored in a single .csv file and distributed to additional members of the research team, including a computational data analyst and behavioral research scientists.

#### Sample

We were broadly interested in the function and utility of The Phoenix app to build community and promote recovery behavior. Given our outlined research questions and project aims, we sought to analyze a robust collection of randomly allocated individual posts originating from The Phoenix app. The dataset included a random collection of 19,685 app entries posted within a 6-month period from October 30, 2023, to April 3, 2024. This sample included 3 endpoints: text ID, anonymized author ID, and post text. After removing duplicates, nonsensical data, and posts comprising random strings, we retained a final sample size of 17,617 posts, which we further processed through our computational pipeline.

### Ethical Considerations

Because this study involves the analysis of deidentified and secondary data, it was deemed as nonhuman subjects research and did not require further review by the institutional review board at Indiana University Bloomington (#24091). Given the sensitive nature of the data, preliminary findings from this study were shared with The Phoenix app members through open-access listening sessions. Any excerpts published in this report have been abridged slightly to uphold app member privacy by removing potentially identifiable information, graphic depictions of substance use, and other personal details.

### Analyses

#### Overview

We used unsupervised NLP and deep learning methods to compositely analyze all posts included for analysis. The analyses used in this study include the Bidirectional Encoder Representation from Transformers topic modeling tool (BERTopic) for theme generation and a Valence Aware Dictionary and Sentiment Reasoner (VADER) sentiment analysis as an approximation for mood and well-being. Both computational analyses are briefly explained in the next sections.

#### BERTopic Tool

BERTopic is a state-of-the-art deep learning topic modeling tool that approximates themes from a collection of similar texts or corpora [33]. Unlike conventional topic modeling analyses, which include latent Dirichlet allocation and latent semantic analysis, BERTopic uses embeddings calculated from a neural network architecture that compares an input sequence against a pretrained large language model [34]. Embeddings calculated by BERTopic are then used to convert the input sequence into continuous vectors, the dimensions of these vectors are reduced, and text-clustering algorithms help extract the most salient themes or ideas in the data. Although topics derived from BERTopic are, in most cases, conceptually clear, human interpretation can help address potential gaps and misclassifications. As such, a manual review of computer-derived topics is recommended to both ascribe meaning to the topics and attain agreement on the meaning among research personnel. For more information on BERTopic and its application in social media user–generated data, refer to Edinger et al and Valdez et al [35,36].

#### VADER Analysis

VADER is a rule-based sentiment analysis lexicon commonly applied to social media text data to measure its valence or affect [37,38]. VADER scores can range from highly negative (−0.99), as in “everything is terrible right now,” to highly positive (0.99), as in “this is the best day of my entire life!” Despite being an older sentiment analysis tool, VADER offers several advantages, including its low computational power requirements and wide range of possible scores, which may reflect a broader range of emotions. These scores can be applied in various general linear modeling methodologies, such as regression and ANOVA. For further details on VADER, the VADER lexicon, and the validation of VADER, see the following [39].

#### Text2Emotion

While VADER is widely regarded as an effective tool for sentiment analysis with social media data, we also ran a Text2Emotion analysis—a similar sentiment analysis tool that captures 5 discrete emotion categories: (1) Happy, (2) Sad, (3) Angry, (4) Surprise, and (5) Fear—for nuance. Scores along these 5 emotions are normalized values from 0 to 1, summing to 1 for each text entry, and reflect the intensity of each measure emotion. VADER and Text2Emotion scores can be compared to substantiate each analysis.

### Procedure

**Figure 1.**
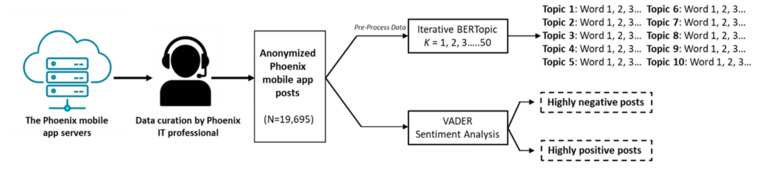
Computational pipeline from data allocation to topic modeling and sentiment analysis. BERTopic: Bidirectional Encoder Representation from Transformers topic modeling tool; VADER: Valence Aware Dictionary and Sentiment Reasoner.

The steps followed in this study are shown in Figure 1. We first preprocessed the data to ensure standard and consistent entries. For our BERTopic analysis, preprocessing steps included removing parts of speech with no clear semantic meaning, such as (1) articles, (2) prepositions, (3) punctuations, (4) abbreviations, (5) any special characters, and (6) numbers. After preprocessing, we ran iterative BERTopic analysis, testing a range of possible topic solutions (k=5…50) to find an optimal topic number. We calculated a coherence score with each iteration as an indicator of model fit, where coherence scores reflect the human interpretability of a fixed set of topics. For each iteration, we plotted each coherence score, with the highest coherence score indicating the optimal solution. Upon running 50 topic iterations, the topic solution with the highest coherence score was 10 (coherence=0.48). Once we identified a topic solution, we applied UMAP and HDBSCAN to reduce dimensions and extract topics with key words (eg, topic 1: Word a, Word b, Word c…Word d). We then used a simple sorting function to match each app entry to a topic based on the overlap of words used in the entry to topic key words. After sorting our topics, the members of the research team met briefly to agree on an interpretation of each topic.

For the VADER analysis, we fed minimally processed text through the VADER lexicon, retaining stop words, punctuations, numbers, and special symbols and characters. Because VADER requires exact-case matching to return an accurate score, that is, VADER can only provide a score if the spelling is correct, we used TextBlob and a loop function to replace apparent typos such that “angryyyy” is automatically corrected to its apparently correct spelling of “angry.” Once each entry received a VADER score and Text2Emotion profile, we created subdatasets consisting of highly negative entries (−0.99 to −0.50) and highly positive entries (0.50 to 0.99), which were reviewed by the research team to ascertain themes.

## Results

### Overview

Broadly, our topic modeling analyses found various topics specific to promoting social connection; our sentiment analysis revealed how people utilize the app to seek or provide social support to those struggling with their recovery journeys. We present the findings from our analysis below.

### RQ1: Using Natural Language Processing and Deep Learning, What Themes Emerge From a Corpus of Posts Originating From The Phoenix App?

**Figure 2.**
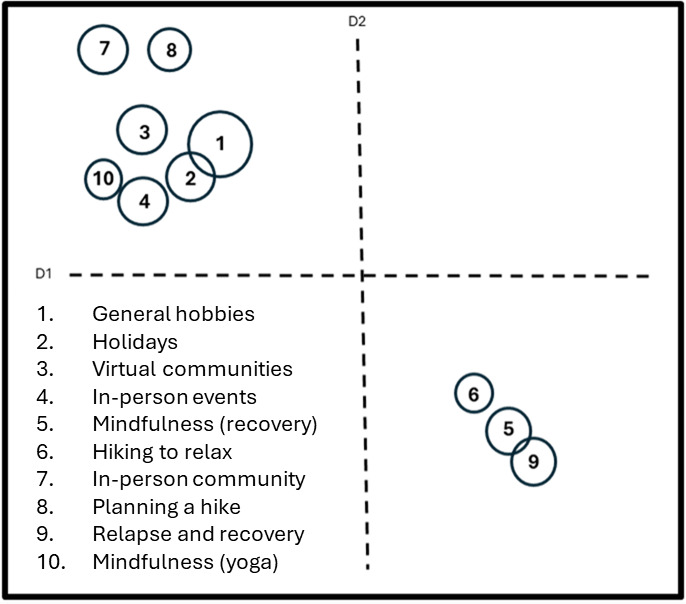
This visualization is an intertopic distance map highlighting the relative similarity between the topics. Around 7 topics appear in the upper left-hand quadrant, and 3 topics appear in the bottom-right quadrant. This suggests 2 overarching themes within the data.

From the iterative BERTopic analysis, it was determined that 10 topics represented the optimal solution with a coherence score of 0.48. We then extracted top terms per topic and their representation as a whole number and percentage of the larger corpus (Table 1), and based on topic keywords, we created a custom name for each of the 10 topics. Topics extracted from our BERTopic analysis revealed what appeared to be 2 overarching themes: (1) a group of topics pertaining to engaging members through in-person and online interactions (7 topics) and (2) as a forum to discuss more serious topics pertaining to substance use and mental health, including struggles with sobriety, relapse, and strategies to promote substance use reduction or abstinence and improve mental health (3 topics). These overarching themes are again observed in Figure 2, which visualizes an intertopic correlation as an intertopic distance map. Dense and closely knit circles (ie, topics) indicate high topic similarity, while disparate topics represent dissimilarity. The topics in the upper left-hand quadrant of the intertopic distance map largely pertain to app dialog about promoting in-person and online events, which ranged from hikes (eg, “Don’t forget about The Phoenix hike tonight at 8 pm!”), workouts (eg, “See you tonight at...the gym for a great workout session”), online and offline poetry sharing (eg, “I am pasting my poem here; let me know what you think”), and other events deemed part of community building among app members. In contrast, the topics in the bottom-right quadrant, while sharing some overlap with the topics in the upper left quadrant, largely pertained to sharing personal experiences; narratives; or insights into substance use, recovery, and substance relapse.

**Table 1.** BERTopic^a^ summary table with 10 topics, top terms per topic, post count (n=17,617), and percentage of the larger corpus^b^.

ID	Custom name	Top terms	Post count (%)
1	General hobbies	favorite - movie - house - film - kitty - best - loved - baby - drew - lil	3417 (19.4)
2	Holidays	holiday - thanksgiving - weekend - Sunday - morning - celebrate - Christmas	1925 (10.9)
3	Virtual community	meet - connect - chat - joined - meeting - event - online - poem - site - join	1826 (10.4)
4	In-person events	festival - Saturday - Wednesday - weekend - fest - event - Thursday - tonight - attend	1785 (10.1)
5	Mindfulness	meditation - prayer - recovery - soul - felt - self - life - care - gratitude - change	1621 (9.2)
6	Hiking to relax	hike - stroll - walk - park - hiking - canyon - trail - leashed - sobriety - trip	1543 (8.8)
7	In-person community	sober - rehab - sobriety - meet - community - recovery - meeting - join - phoenix - hi	1491 (8.5)
8	Planning a hike	hike - hiking - trail - location - nj - area - az - hiker - valley - boulder	1447 (8.2)
9	Relapse	relapse - rehab - relapsed - substance use - addict - sobriety - sober - recovery - drug - stay	1365 (7.8)
10	Mindfulness	sobriety - recovery - walking - fitness - walked - yoga - volunteer - daily - exercise	1197 (6.8)

a BERTopic: Bidirectional Encoder Representation from Transformers topic modeling tool.

b Per topic, we extracted the 10 most salient terms. These terms were used to match app entries to a corresponding topic. The most prominent topic in our data, “general hobbies,” comprised 19.4% (n=3417) of all entries, while the least prominent topic was “mindfulness,” comprising 6.8% (n=1197) of all entries.

Upon reviewing topic keywords and intertopic similarities, we then used a sorting function to sort posts into one of the 10 possible topics based on keyword matching. From this sorting, we were able to identify a series of optimal quotations, which added further context and nuance to the topics explained above (Table 2). Table 2 shows the actual conversations unfolding with greater nuance from the dataset.

**Table 2.** Representative excerpts from the dataset per topic, parsed by topic ID, topic name, and key excerpts^a^.

Topic ID	Topic name	Key excerpts
1	General hobbies	*Hey beautiful people. What an amazing way to connect. I need new friends in the area. My hobbies: art, music, hair, meetings (lol!), beach!* *Its honestly been so long since I have done anything social, so I am just figuring out what my hobbies and things I like to do are!* *Hi friends! Looking to build a strong sober friend group. I have lots of hobbies, passions and ideas for groups. Let’s be friends!*
2	Holidays	*Merry Christmas and Happy Holidays from our family to yours!* *9am X-mas Eve X-fit crushed it this morning! Happy holidays to all! Come out and join us in the fun next time!* *I’m feeling a bit stressed with the Holidays coming up. I’m looking to chat with a new social group to help*.
3	Virtual community	*Hey Phoenix moms! I’ve found that sharing stories of recovery and resilience, can break down stigma and improve community.* *Happy to find a new online community in this new journey! Shine On!* *Good morning!! finding this new community has given me hope that staying connected and making friends will help me stay on course*.
4	In-person events	*Tonight I attended a first of its kind, local, in-person event that brought together feminine energies in a very chill, vulnerable, and empowering way.* *Thank you to everyone who showed up last night to my event!!! It was a blast to see some returning faces, as well as a ton of new faces!* *Hi! Would love to meet the Phoenix community here in my area*
5	Mindfulness (recovery)	*Today I was mindful of my body and breath during my meditation. I also focused on being more mindful of my surroundings.* *Hey! How do you bring more mindfulness into your life?* *Mindfulness is such a big help in my recovery. When I start getting negative thoughts I’m able to bring my mind back to the present*.
6	Hiking to relax	*Hey guys. Anyone use hiking to relax and get away? Let’s connect!* *looking for some relaxed hiking partners in [REDACTED]. Check out our hiking schedule and come relax with me!!!*
7	In-person community	*Last night, we had a party for the wonderful folks who offer their time, energy, and passion to our community. Drop a comment and show them some love!* *Hey everyone! Starting May 2nd we will be doing a month long community challenge all about meditation and mental health*. *New to the sober community! Need to find a sponsor and supportive group*.
8	Planning a hike	*Is there a [REDACTED] hike coming up soon? I’m thinking about going in the morning but wasn’t sure if there was already a planned hike. Thanks!* *Come join us for a hike this Saturday.* *We hike regularly on Sundays if anyone is interested let me know!!!!*
9	Relapse and recovery	*Trying to accomplish sobriety momentum. Having trouble getting started after a recent relapse.* *Unfortunately I relapsed. Today is my first serious step in my road to recovery.* *I lost my sobriety on Thursday & I relapsed again yesterday*.
10	Mindfulness (yoga)	*I am interested in learning to teach yoga and meditate any recommendations on how to do this? Thx.* *Yoga and meditation was awesome. Motivational and totally worthwhile. Appreciate all the volunteers, staff and instructors. Thank you.* *However, I am back to it today, I did about 20 minutes, very gentle. My anxiety was really high today, and yoga and meditation really did help*.

a Note that the discourse appears to contain a mix of app-member and app-volunteer dialog. All excerpts reported in this table have been slightly abridged to uphold the privacy and anonymity of app members by removing personal details, locations, and graphic depictions of substance use.

The excerpts in Table 2 highlight representative themes, discussions, or comments within our sample. These excerpts were identified using a sorting function that categorized app posts into one of the 10 topics identified by the BERTopic tool. These quotes demonstrate a distinct and recurring theme of community support. This support manifests through encouragement, the promotion of online activities, and in-person events, including hiking, physical activity, mindfulness, meditation, and other similar activities that promote recovery through movement and community engagement.

### RQ2. What Do Compound Sentiment Analysis Scores Imply Regarding Personal and Collective Experiences of Phoenix App Members?

To further substantiate the topic modeling findings, which were used to identify recurring themes in the social media entries collected from the dataset, we applied VADER sentiment analysis to all collected entries, excluding duplicates and retaining “zero” values. Table 3 provides an overview of our findings, highlighting the overall mean VADER score for the entire sample, with a possible range of −0.99 to 0.99. It also includes a summary of highly positive entries—those scoring above 0.50—and highly negative entries—those scoring below −0.50.

**Table 3.** Descriptive VADER^a^ findings overall and stratified by highly negative and highly positive entries^b^.

Category	Mean VADER score (SD)	Median VADER score (IQR)	Quartiles of the positive VADER scores			Sample size, n (%)
			0.25	0.50	0.75	
Overall data	0.44 (0.42)	0.54 (0.83)	0.00	0.54	0.83	17,922 (100)
Highly negative posts	Below −0.50	—^c^	—	—	—	429 (2.4)
Highly positive posts	Above 0.50	—	—	—	—	9363 (52.2)

a VADER: Valence Aware Dictionary and Sentiment Reasoner.

b Within the overall data, there is a right skew, indicating a greater presence of highly valenced, or positive, entries. The median and quartile ranges likewise suggest a high presence of “0.00“ VADER values, which comprise neutrally valenced, or nonemotive, entries (eg, “Meet at the park today?”), or very short entries that were not scorable with the VADER lexicon (eg, “Hi”).

c not applicable.

The mean VADER score of our entire sample, after eliminating duplicate entries, was 0.44 with an SD of 0.42. This suggests that, overall, valence, or affect, was highly positive with notable variation across all entries. The median and quartile regions, however, strongly imply a right-skewed distribution, implicating higher levels of positive content. When we further subdivided our sample to extract highly positive and highly negative entries, we observed that over half (52.2%) of our sample contained VADER scores greater than 0.50. Conversely, only 429 entries, or 2.4% of our data, scored below –0.50.

To validate our VADER findings, we ran a Text2Emotion analysis with the overall data. Unlike VADER, which provides a single composite score that ranges from –0.99 to 0.99, Text2Emotion measures 5 emotion categories: (1) Happy, (2) Sad, (3) Surprise, (4) Angry, and (5) Fear. It then scores them as normalized values that sum to 1 for each entry. These scores reflect the intensity of each emotion expressed. Across all entries, “Happy” was the most prominent emotion (mean 0.25), followed by “Fear” (mean 0.19), “Sad” (mean 0.17), and “Surprise” (mean 0.16), while “Angry” was the least prominent emotion (mean 0.04). As with VADER, we noticed some potential misclassification (eg, “That workout killed me today“ scored high for “fear”). However, the findings from VADER and Text2Emotion both implicate the presence of highly positive content.

Table 4 provides 5 abridged excerpts of highly negative and highly positive posts. From these excerpts, we observed clear differences in the content. For highly positive excerpts, we observed people expressing gratitude for their sobriety and mental health; appreciation for The Phoenix, the community, and other support structures; and promotion of in-person and online events available in locations across the United States. As seen in Excerpt: “I am just so thankful to be alive today” and “Just had a great workout, make sure you stop by if you are in the [redacted] area!,” the presence of positive language with words including “thankful,” “alive,” “great,” and phrases such as “just had a great workout” contribute to higher than average VADER scores. Conversely, negative entries seemed to exhibit 1 recurring theme—individual narratives about struggles with sobriety, relapse, mental health, or voicing needs for additional support.

It is also worth noting that some negative entries concluded with words of encouragement, as seen in the excerpt: “If I can get through it, so can you.”

**Table 4.** Excerpts from highly negative and highly positive posts from the dataset^a^.

VADER^b^ subset and key quotations		Score
Highly positive excerpts		
I’m grateful for new experiences in my life. I’m grateful for therapy. I’m grateful for support.		0.99
What’s on my mind? I’ll begin with giving thanks to the lord above for the breath in my lungs and to rebuild something better for my future.		0.99
I got to see a recovery milestone today and that means a lot to me.		0.99
I didn’t go out last night, woke up this morning feeling ready to tackle the day. I wish you all a wonderful, peaceful, exciting Sunday.		0.98
[REDACTED] has teamed up with 1 Million Strong, an impact initiative that seeks to transform the way people think about addiction and recovery and The Phoenix, a National Sober Active Community, which fosters a community for those in recovery to host a sober-supportive wellness tent experience for festival-goers to cool off, relax, and enjoy alcohol-free cocktails in a comfortable environment.		0.98
Highly negative excerpts		
I know I don’t want to but I need a clean and sober place to live out of the city. I go to meetings, I try and stick with the winners and yet I still find myself giving into my triggers to use. I really hate asking for help, and I find it burden others who are in recovery as well.		−0.99
Not doing well at right now. Current circumstances make it near impossible for me to get my treatments.		−0.99
I do not feel like I am up for this this. Using has taken control of what life I have left and I wish every night that the sun didn’t rise. im lost and I am sad.		−0.98
Trying to get sober again after a difficult relapse that has kept me in addiction the past eight I am feeling alone and scared.		−0.96
I had to cancel attending a social gathering tonight. I felt so bad about but I am just exercising my boundaries because I do not like being in situations where people drink. Its ok to say NO when and if it feels right. A sober mind & body can hear itself.		−0.94

a Note that to uphold the privacy and confidentiality of the app members, these excerpts have been slightly abridged to remove any personally identifiable information; specific details; or graphic depictions of drug use, struggles, or relapse.

b VADER: Valence Aware Dictionary and Sentiment Reasoner.

### RQ3: How Can Data Mining Strategies Inform Future Adaptation of The Phoenix App and Member Experiences?

Our study used topic models and sentiment analysis to examine recurring themes, uses, and valences of randomly selected entries from the dataset. Using NLP, it is possible to increase the granularity of analysis to triangulate phrasings counterintuitive to recovery. Using sentiment categories and strategic queries, we attempted to allocate content that may imply highly negative experiences, warranting response or intervention. For example, we queried the data to isolate all entries containing the phrase “I need help,” which also contained a negative VADER sentiment value (ie, values between –0.01 and –0.99). We also searched a few additional phrases that, in the psychology literature, indicate personal rumination, worry, or distorted thinking, including “I am a”—a type of distorted thinking characterized by labeling oneself and “I will never”—a type of distorted thinking characterized by overgeneralizing or fortune-telling. We hypothesized that people using these phrases in a negative context reflected some distress or worry, should they appear in the dataset.

Although these queries returned very few entries, as seen in Table 5, they illustrate how some individuals used the app to seek support from other app members. In this context, seeking support reflected a general struggle with one’s current recovery journey. At the same time, we observed individuals posting poetry, which may not directly reflect personal experiences with substance use, mental health, and recovery.

**Table 5.** We used strategic queries to identify posts that may indicate when someone is reaching out for help and assistance from the dataset^a^.

Query	N	Excerpt	VADER^b^ score
I need help**…**	2	I’m so stressed out Things could be great for a while I seem to have a handle on things but all of a sudden it’s like my brain shifts quickly into the old way of thinking I don’t know where it comes from in that moment of time I freeze without a thought I have lost myself I can’t breathe I can’t think clearly and I panic. My mind floods with thoughts of negativity I need help I want to live a meaningful life and start healing completely to move forward I’m here because I want to be I don’t know what I’m doing.	−0.47
		I want to shout but I’m fine comes out Trapped in my own head I’m confined. I need help!	−0.97
I am a…	17	This is getting to me I work nights and really going through depression. I got put on meds to not drink that’s good-- well I am feeling very alone and can’t really get to the outings. I hate feeling like my world is over and not caring about anything anymore it’s not me I am an awesome person and need to get out of this rut or find some hobbies.	−0.86
		Hi I am an alcoholic and I have been drinking a lot. I do it to quiet my brain at times.	−0.54
I will never…	5	Today the prettiest girl in school died Killed when my car hit another and burned to the ground I knew I was drunk yet I still had to drive Tell them I wish I could have stayed around. I know I cannot go back and give up the keys. I am not going to be there when my baby learns to walk. When she falls down and needs me to bandage her knees, I won’t ever kiss away life’s pain OH GOD I will never hear her talk.	−0.95
		Yesterday marked 6 months for me. I remember not being able to make it past 4 days! Anyone have any advice to help cravings? I will NEVER go back but the cravings get so bad I get depressed. Thank you!	−0.52

a While our search did not retain much data, we observed that these queries consistently returned representative data of sobriety struggles. Please note that to uphold the privacy and anonymity of Phoenix app members, these excerpts have been abridged slightly to remove personally identifiable information, locations, and graphic depictions of substance use.

b VADER: Valence Aware Dictionary and Sentiment Reasoner.

## Discussion

### Principal Findings

This study tested a computational NLP pipeline to analyze randomly allocated posts collected from The Phoenix app. Broadly, our findings suggest that app members use the app to promote social connection and community. These findings align with a growing body of literature indicating that mobile apps are effective systems for delivering behavior change interventions. We provide additional context below.

### High Levels of Positivity and Inclusive Dialog on The Phoenix App May Demonstrate How Closed Social Media Platforms Differ From Conventional, Open Social Media Platforms Regarding Recovery

Numerous studies use VADER as a proxy for public mood or subjective well-being and as a primary measure to understand social attitudes expressed on social media during major public health events [38,40-42]. These studies typically involve the composite analysis of very large collections of posts from various social media platforms such as X, Reddit, and YouTube, often around a singular fixed topic ranging from substance use and recovery [38], reproductive rights and access [43], collective mental health [44], and others.

Although our study employs a similar use of VADER (ie, as a tool to measure the mood of text data), we observed key differences in our findings that diverge from previous studies. Indeed, prior studies using VADER sentiment analysis in both health and social media contexts sometimes report a mean VADER score near 0 [43]. Distributions of these VADER scores are commonly bimodal [45], indicating a near-equal presence of highly negative and highly positive content. When calculating a mean VADER value, this bimodal distribution can sometimes yield a mean VADER value near 0 with a high standard deviation, making it challenging to draw reasonable conclusions about collective mood, well-being, or valence regardless of the topic—though more research on this topic is needed that addresses VADER limitations is needed.

However, in our analysis of over 17,000 recovery-related entries, we observed a mean VADER score of 0.44, reflecting a generally high presence of positive content. A more detailed review of the descriptive statistics revealed that the VADER score distribution was not bimodal but positively skewed. This finding is further supported by the relatively low number of highly negative posts (ie, those scoring lower than –0.50), which comprised just over 2% (n=429) of the corpus. In contrast, highly positive posts (ie, those scoring higher than 0.50) comprised more than 50% (n=9363) of our sample.

The general positive tone uncovered by VADER is further supported by the Text2Emotion analysis—where “happy” was the most dominant emotion—and themes uncovered in our BERTopic analysis. Some themes broadly referenced various activities and interests, while others appeared to originate from volunteers marketing specific online and in-person events to the membership. These themes may implicate online social connection, which can drive people to continue using the app as part of their recovery process.

Critically, we observed 1 topic that referenced relapse and sobriety struggles. This topic illustrates how, even if in very small quantities, people are willing to discuss their struggles openly, which we interpreted as an effort to seek support. Collectively, these topics offer evidence that, as a recovery smartphone app, curated app experiences may offer more directed discourse needed to support and sustain recovery. Future research should compare these findings with data from other similar recovery apps to provide further evidence that digital tools offer cost-effective approaches to promoting recovery regardless of geographic access.

### Topical Recovery Content With High Sentiment Values Illustrates the Need for Tailored and Safeguarded Social Media Experiences

In recent years, a growing body of literature has explored social media’s role in promoting social connection, particularly in relation to health and well-being [32,46-48]. The findings from that work highlight that the degree and quality of social connection tend to vary and often depend on the specific platform, medium of the platform, and context.

There are numerous examples of how social media can facilitate social connections. Studies on Facebook groups have found that the degree of connection offered was particularly beneficial for older adults [47] and non-native English speakers [49]. These groups are also the most dominant Facebook user base [49]. On X, before the 2022 acquisition by Elon Musk, people could search for communities by grouping related content through hashtags, which created pockets of conversation around specific topics. Reddit remains widely regarded as one of the strongest conventional social media platforms for fostering social connections through curated subreddits with trained moderators [50]. However, recurring issues across these platforms remain, such as low-quality health information, trolling, and exposure to harmful content counterintuitive to recovery [51].

Prior studies have explored the strengths and weaknesses of social media for recovery using conventional social media data, such as X or Twitter, including how communities form around topics such as alcohol abstinence during Dry January [52] and how others communicate about personal and familial drug use online [53]. While platforms such as Facebook, X, and Reddit have been leveraged to connect people with shared experiences, their general nature may not always yield positive experiences. This is potentially due to the overwhelming number of unique posts across a wide range of topics, a majority of which do not pertain to health and well-being, or inconsistent moderation that filters negative content.

To offset negative social media interactions, some argue that the approaches to safeguard spaces via social media–like mobile apps can create a curated social media experience tailored to specific needs, thereby enhancing both the quality of perceived social connection and perceived benefits of support communities [54,55]. The Phoenix app exhibits the characteristics of effective safeguarding through advanced monitoring and interactions between members and volunteers. The more curated nature of the app—designed with the express purpose of connecting people in recovery—allows for a more directed experience that reaches its intended audience.

Further evident in our data, which included topics related to social interaction and recovery, and sentiment analyses indicating high levels of app positivity, the app displays early signs of a supportive and balanced social media ecosystem where safeguarding is effective. By safeguarding the app, app members are protected from incongruent content that may be encountered on conventional social media platforms, creating a safer experience that builds trust among app members and volunteers.

### Continued Push Toward Novel Strategies to Improve the Experiences of Online Recovery Communities and Their Impact

Our findings suggest that digital recovery apps can promote a sense of community and belonging among people in recovery from substance use. To substantiate these observations, we applied an unsupervised NLP framework, which has been used in previous studies to contextualize how people express themselves about certain health topics or leverage online communities for social support or information seeking [32]. Collectively, these findings contribute to prior work calling for directed app experiences for behavior change, though additional research could add further context to our findings to address potential challenges associated with social apps designed with specific populations and objectives in mind. Still, additional work with and beyond the Phoenix is necessary.

Future research should strongly consider the following. First, The Phoenix app is distinct from other social media platforms in its emphasis on safeguarding and advanced monitoring to filter harmful content. While our sample did not identify any content deemed counterintuitive to recovery, further analysis of larger Phoenix datasets could provide deeper insights into the effectiveness of these processes—particularly for app members early in recovery or other vulnerable groups who are likely at a greater risk of negative effects from engaging with triggering or harmful content (ie, veterans and LGBTQIA-identifying members). From a cross-disciplinary perspective, additional work with other computer science fields, including machine learning and artificial intelligence, can help develop additional tools to directly intervene when someone posting on The Phoenix app writes in distressing tones. Qualitative interviews and codesign sessions with app members can also help explore personal experiences with the app’s safety and support features, especially when compared with other mobile apps with a similar focus. Critically, any insights generated from that work would accomplish 2 tasks: (1) establish software-specific best practices for recovery-related mobile apps and (2) identify nuances in recovery communication across online versus conventional, residential programs.

Second, while our analysis points to the success of the app in fostering a supportive recovery environment, our findings have only been compared to prior studies using conventional social media data on substance recovery. Future studies should consider conducting comparative analyses of recovery forums across curated recovery apps, specifically using both qualitative and computational methods. An across-app comparison could add additional validity to our findings, particularly regarding the relative strengths of resources and tools offered by the Phoenix relative to tools and resources offered on apps from other recovery groups and organizations. These additional research directions will strongly contribute to our understanding of smartphone apps’ roles and functions as promoters of positive behavior change and lend further evidence to optimal smartphone app designs to promote sustained engagement in recovery.

### Limitations

This study is subject to limitations we hope to address in future work. First, our study involved a cross-sectional analysis of randomly allocated posts derived from The Phoenix app. While our findings offer preliminary insights into the use and purpose of the app, it is likely that our data are not entirely reflective of in-the-moment discourse that may demonstrate other app utilities and uses. For example, the dataset contained specific channels or communities directed at particular topics and experiences, including pets, art, and others. Although it is possible our data may be overrepresented by one or a few channels, it is not currently possible to assess this with the specific sample. We strongly encourage future research to use our pipeline with additional Phoenix data that removes, or at least compares, volunteers to app members. Second, we acknowledge that our NLP approach is entirely unsupervised, meaning no training data were leveraged. While this may limit the novelty of our approach, we contend that the analyses used provide a strong scaffold that is necessary to design, program, and ideally, implement more sophisticated tools for recovery, including knowledge graphs, retrieval-augmented generation systems, and fine-tuned large language models. Third, we also acknowledge that, at the time of writing, The Phoenix app has been rebranded as NewForm. Although The Phoenix app and NewForm are similar in functionality, it is likely that newer features may result in marginal variations in our findings.

### Conclusions

This study tested a computational pipeline aimed at contextualizing the digital environment of a recovery-focused app developed by The Phoenix, a national nonprofit with over 65,000 monthly active members. Notably, our pipeline aimed to extract language, emotions, and behaviors expressed by app members to ascertain app use and its impact in fostering recovery support in a digital environment. These findings—which illustrate the broadly positive uses of an app as evidenced by the themes and emotions expressed by app members—add further evidence to the growing body of research calling for online recovery resources to address the chronic shortage of costly residential treatment options. Further, these findings likewise implicate key recovery dynamics in digital spaces—including how people in active recovery discuss their journeys, support one another, or share resources. Extracting and modeling these insights can further aid in the design of more robust tools aimed at keeping people in recovery by meeting them where they are in their journeys.

## Abbreviations

BERTopic: Bidirectional Encoder Representation from Transformers topic modeling tool
NLP: natural language processing
RQs: research questions
SUD: substance use disorder
VADER: Valence Aware Dictionary and Sentiment Reasoner
